# Servant leadership and employee voice behavior: the role of employee work reflection and employee proactive personality

**DOI:** 10.3389/fpsyg.2024.1421412

**Published:** 2024-07-29

**Authors:** Zelei Xu, Yu Gu, Hongyu Wang, Lili Liu

**Affiliations:** ^1^School of Economics and Management, Changchun University of Technology, Changchun, China; ^2^School of Business and Management, Jilin University, Changchun, China; ^3^School of International Economics and Trade, Jilin University of Finance and Economics, Changchun, China

**Keywords:** servant leadership, employee voice behavior, employee work reflection, employee proactive personality, social cognitive theory

## Abstract

Despite the recent proliferation of scholarly investigations on servant leadership, clarity remains elusive regarding the specific mechanisms and conditions underpinning employee cognitive processes and their responses to servant leadership. Drawing upon social cognitive theory, proposes a moderated mediation model tested through a time-lagged field data from 489 employees in Study 1 and an experimental data in Study 2. We found that servant leadership indirectly enhances employee voice behavior through increased employee work reflection. Additionally, we considered employee proactive personality as a boundary condition for the positive effect of servant leadership. Our results show that servant leadership prompts employee work reflection only when the level of employee proactive personality is high, which in turn increases employee voice behavior. This study presents significant theoretical and practical implications through the integration of social cognitive theory with servant leadership research.

## Introduction

As organizational environments become more diverse, complex, and dynamic, the importance of upward information flow and multi-source perspectives within organizations has become increasingly vital for effective decision-making and overall organizational health (Morrison and Milliken, 2000; Li and Tangirala, 2022). In this context, employees’ value to enterprises extends beyond their labor contributions to include their ability to generate innovative ideas and viewpoints (Morrison, 2011; Welsh et al., 2022). Consequently, employee voice, defined as the expression of work-related opinions, ideas, and concerns driven by cooperative motivation, has become increasingly important for organizational success (Morrison, 2014; Parke et al., 2022).

However, employees often perceive voicing their opinions as risky (Dutton et al., 1997; Zhao et al., 2023). Publicly expressing views on work-related issues can disrupt organizational harmony and challenge leadership authority (Milliken et al., 2003). This can not only fail to influence management decisions but also potentially have negative repercussions for career development (Parke et al., 2022). Given the importance of employee voice and the ambivalence felt by employees when expressing their opinions, the role of leadership in influencing voice behavior has become a central focus of research (Jiang et al., 2022; Thompson and Klotz, 2022). In particular, servant leadership, characterized by service, altruism, and empowerment (Kauppila et al., 2022; Li et al., 2023), has garnered significant attention from researchers for its potential to promote employee voice behavior (Greenleaf, 1977; Liden et al., 2014). Central to this leadership style is its focus on understanding and fulfilling the individual needs of employees, appreciating their unique values, and fostering their participation in servant behaviors (Greenleaf, 1970). The direct encouragement of voice behavior underlines the unique position of servant leadership in enhancing open communication and driving innovative changes within organizations (Detert and Treviño, 2010; Arain et al., 2019; Liao et al., 2021; Hartnell et al., 2023). Prior research has demonstrated that servant leadership is favorably correlated with followers’ positive extra-role behaviors (Ehrhart, 2004; Lapointe and Vandenberche, 2015; Sun et al., 2019; Wu et al., 2021).

Although it is anticipated that servant leadership will boost employees’ voice behavior by providing a work atmosphere that is relationally friendly, employees engage in complex cognitive processes before deciding to voice their opinions (Bandura, 1986, 1991). These cognitive processes reflect employees’ evaluation of the potential positive and negative outcomes of their voice behavior (Yang et al., 2022). Existing research seldom investigates the mechanisms and conditions under which humble leadership influences voice behavior through cognitive processes (Ong et al., 2023). According to social cognitive theory (SCT), the self-regulatory function is performed by self-evaluative reactions resulting from an individual’s self-cognitive capability and established internal standards. These, in turn, serve to influence both cognition and action (Bandura, 1986, 1991). As a core mechanism of regulation and key predictor of employee behavior (Schippers et al., 2015), employee work reflection has received considerable attention. Employee work reflection refers to the voluntary involvement of an individual in a series of cognitive processes, wherein they contemplate and analyze many aspects that constitute their work and influence their capacity to get favorable work results (Ong et al., 2023). Even though various cognitive factors may impact the consequence of servant leadership, individual work reflection may be the most significant core mechanism and key predictor of leadership effectiveness (Li et al., 2020; Lanaj et al., 2023; Ong et al., 2023). Specifically, employee work reflection is intimately connected to the core attributes of servant leadership: This environment of trust and collaboration, characterized by an emphasis on listening, empathy, and stewardship, is essential, as it brings employee reflection to the fore (Walumbwa et al., 2010). Employee reflection arises from the support and psychological safety provided by the external environment. In addition, servant leadership embodies the management philosophy of being “employee-centered” and shows a high degree of interpersonal acceptance and willingness to serve (Greenleaf, 1977; Liden et al., 2014). This bottom-up leadership approach enhances the employees’ sense of belonging and pride in the organization, which helps them to minimize the risks associated with voice behavior through reflective thinking (Leblanc et al., 2022).

**Figure 1:**
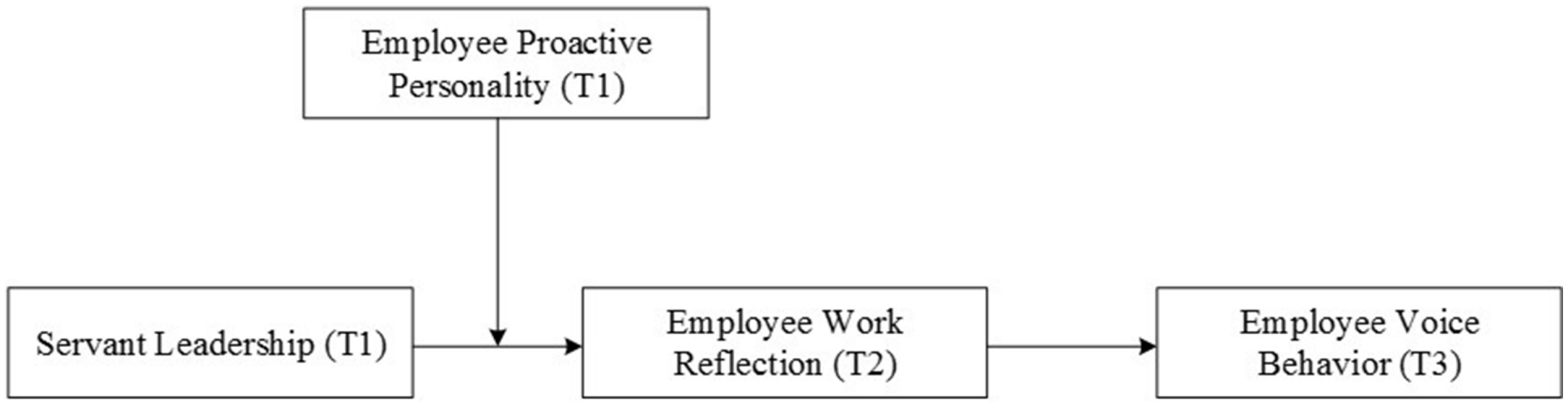
Conceptual model.

Yet, the effectiveness of servant leadership in influencing individual employee behavior remains uncertain, as these complex cognitive processes can be significantly affected by individual differences. Previous research suggests that employees with a proactive personality, characterized by high self-efficacy and resilience, are more likely to exhibit increased motivation. In contrast, employees with less proactive personalities may not experience the same perceptions (Li et al., 2020; Wu et al., 2021). As a result, servant leadership may only materialize into employee work reflection and ultimately employee voice behavior under certain conditions. In SCT, “triadic reciprocity” highlights the interactivity of individual traits with which the organizational environment influences individual cognition and behavior (Bandura, 1986). Specifically, we argue that the effectiveness of servant leadership can vary greatly depending on employee proactive personality. Proactive personality is defined as an individual’s behavioral inclination to proactively adapt to the external environment and to positively change the situation (Crant and Bateman, 2000). Given that an employee with a highly proactive personality is able to make full use of their subjective initiative, they are inclined to exhibit heightened attentiveness toward servant leadership. In effect, they perceive servant leadership to be a means to bring about self-value (Li et al., 2020). In particular, this study proposes and demonstrates that employees who exhibit proactive tendencies are more likely to leverage the work environment fostered by a servant leader in order to engage in work reflection. In contrast, less proactive employees are more inclined to stick to their current work and are unwilling to seek new breakthroughs before engaging in reflection and adapting their objectives and approaches. Therefore, this study suggests that the interaction between employee proactive personality and servant leadership encourages employee work reflection, which in turn encourages employee voice behavior. In summary, the conceptual model is depicted in Figure 1.

This study makes two major contributions to existing literature. First, SCT is applied as a framework to integrate a distinct cognitive construct into the existing bodies of research on servant leadership and voice behavior. Specifically, that construct is employee work reflection. Employee work reflection complements the forward-looking perspective to delineate how employees engage in voice behavior in response to servant leadership through internal self-reflection. By highlighting work reflection, this study provides a deeper understanding of how cognitive processes influence the way employees perceive and react to servant leadership, thereby offering a more comprehensive view of the mechanisms driving voice behavior in the context of servant leadership. Second, by identifying employee proactive personality as a moderating factor, this study underscores the importance of individual differences in shaping the outcomes of servant leadership. It extends the existing servant leadership literature by elucidating how employee-related factors, can influence the effectiveness of servant leadership. Additionally, this study helps develop SCT in the servant leadership domain by recognizing employee personality as a key compositional factor that can modulate the influence of leaders as role models within their organizations.

## Theory and hypotheses

### Social cognitive theory and theoretical model

This study explains the impact of servant leadership on employee voice behavior according to SCT. Founded by Bandura, SCT is the basic theory of individual behavior; SCT refers to the continuous and dynamic interactive relationship between the external environment, cognitive factors, and individual behavior. According to Bandura (2006), a bidirectional relationship exists between any two factors, and they constantly change under different environments, individuals, and behaviors. An individual’s behavior is seen as a product of the combination of individual cognition and external environmental factors. In addition, SCT has been extensively employed to gain insights into and make predictions about individual behavioral choices and behavioral characteristics (Leblanc et al., 2022).

Within the framework of SCT, servant leadership is characterized by a supportive and empowering essence and serves as a distinct environmental stimulus. Its influence manifests by promoting individual cognitive responses (work reflection and voice behavior) among team members regarding their work roles within the organization. Moreover, proactive personality serves a pivotal role in determining how individual employees exploit this supportive environment. Proactive individuals are hypothesized to exhibit more robust responses to servant leadership, given that their innate tendency to actively engage with their environment bolsters their reflective processes and amplifies their vocal expressions. This interaction highlights the complex interplay between personal characteristics and environmental cues in shaping behavior, reflecting the SCT’s perspective that behavior is a product of both personal and environmental factors.

### Servant leadership and employee voice behaviors

Servant leadership is a style of leadership in which the satisfaction of employees’ needs, desires, and interests are prioritized and used to lead subordinates. The leaders acquire employees’ confidence and exert influence by regularly providing services to them (Ehrhart, 2004; Liden et al., 2008). In essence, servant leaders are primarily motivated by service rather than leadership; they see leadership positions as an opportunity to serve others. Such leaders model the way for subordinates to learn; the ultimate goal of service is to help them grow as servants, thereby benefiting the whole organization (Lemoine et al., 2019; Li et al., 2023). Within this novel context, servant leadership has been proven to be actively correlated with both individual positive behaviors (such as pro-social behavior) and job performance (Eva et al., 2019; Hui et al., 2020). This study posits that, within organizations, servant leadership heightens employee voice behavior. Specifically, SCT emphasizes the dynamic interaction between the external environment and an individual’s behavior. An individual observes the exemplary behaviors of others to construct a self-cognitive awareness and in this way either directly or indirectly learns appropriate behaviors (Bandura, 1986). As an extra-role behavior, voice behavior helps the organization to obtain its objectives and also enhances organizational effectiveness. This is the effective outcome of employee modeling and learning form servant leadership (Walumbwa et al., 2010). At the same time, servant leadership tends to empower and promote self-management by employees, while voice behavior allows employees to express their views and provide suggestions regarding problems in organizational decision-making (Hunter et al., 2013). That is, servant leadership provides a channel through which employees can add their voice. In addition, the empowerment of servant leaders’ advice will stimulate employees’ sense of efficacy and self-confidence, keep them enthusiastic in the work process, and make it easier to motivate them to use their voice (Duan et al., 2014). Thus, the following hypothesis is formulated:

*Hypothesis 1*: Servant leadership is positively related to employee voice behavior.

### The mediating role of employee work reflection

Drawing upon the framework of social cognitive mechanisms, this study maintains that individuals engage in the process of understanding the external environment in order to adjust their self-cognition and behavior. Ultimately, this adaptive process serves the objective of establishing and maintaining consistency with the external environment (Bandura, 1986). In line with this framework, we propose that servant leadership heightens employee work reflection, acting as a cognitive bridge between leadership influence and employee voice behavior. This reflective process includes examining past actions, considering alternative strategies, and planning future actions, thereby enhancing employees’ cognitive and emotional involvement and commitment to their work (Schippers et al., 2015; Lyubovnikova et al., 2017). Previous studies have examined other mediators of the relationship between servant leadership and employee outcomes. For instance, psychological safety has been identified as a key mediator, explaining how servant leadership fosters an environment where employees feel safe to express their ideas without fear of negative consequences (Edmondson, 1999). Similarly, psychological empowerment has been explored as a mediator, highlighting how servant leadership enhances employees’ sense of autonomy and control over their work (van Dierendonck and Nuijten, 2011).

On the one hand, servant leadership, characterized by its service and empowerment, uniquely stimulates employees’ potential and challenges them to enhance their self-efficacy. This leadership style guides the overall development of employees within an atmosphere of service, fostering an environment where positive cognitive processes are initiated (Ong et al., 2023). Further, servant leadership promotes individual reflection by providing a model for service and behaviors that employees can emulate (Zhang et al., 2012). For example, when leaders demonstrate humility and a commitment to service, employees are more likely to reflect on their own behaviors and strive to align them with these values (Walumbwa et al., 2010). This modeling effect triggers a positive cognitive response, strengthening employees’ sense of responsibility and mission within the organization. Consequently, servant leadership enables employees to get more service, support, and resources, thereby stimulating employee work reflection.

On the other hand, as a cognitive state, employee work reflection will further enable employees to get a deeper comprehension of how they can cognitively contribute functional value to their organizations. Prior research has identified work reflection as key for employee organizational citizenship behavior and prosocial behavior because it integrates diverse experiences and enhances awareness of organizational needs and challenges (Carlson et al., 2016; Thompson and Bolino, 2018). By reflecting, employees can evaluate work processes, identify improvements, and develop actionable suggestions, fostering continuous improvement (Schippers et al., 2015). Moreover, positive outcomes from reflection can motivate employees to set more ambitious and innovative goals, encouraging them to invest resources and effort into voicing ideas (Bandura, 1991; Schaubroeck et al., 2011; Lyubovnikova et al., 2017). Thus, employees can use reflection not only to address challenges but also to propose new initiatives, aligning with organizational objectives (Bandura and Locke, 2003; Parker et al., 2010). Furthermore, work reflection enhances employees’ emotional regulation, which is crucial when engaging in voice behavior, often seen as risky (Liang et al., 2012). Effective emotional regulation can reduce the anxiety associated with voicing concerns and suggestions. Taken together, this study proposes:

*Hypothesis 2*: Employee work reflection mediates the relationship between servant leadership and employee voice behavior.

### The moderating role of employee proactive personality

Although servant leadership is typically perceived as supportive and empowering, its effectiveness can vary depending on employee personal traits. For instance, servant leadership has been found to be positively related to employees with higher levels of self-interest (Wu et al., 2021) or political skills (Liao et al., 2021). As has been proven, SCT reveals that individuals process information about the external environment through individual factors, such as typical personality traits, which jointly affect individual psychological cognitions and behaviors (Martinko et al., 2002). Thus, it is crucial to gain a more comprehensive understanding of the specific individual characteristics that determine the effectiveness of servant leadership.

As a key aspect of the individual factors (Maloney et al., 2016), this study suggests that the phenomenon of employee proactive personality can moderate the magnitude of servant leadership effectiveness (Chiu et al., 2016; Hu and Judge, 2017). Proactive personality is defined as an inclination toward initiative and perseverance in effecting meaningful change (Crant and Bateman, 2000). Specifically, employees with a high level of proactive personality tend to engage more actively in a servant leadership climate. Such employees are adept at identifying opportunities and are motivated by the servant leader’s behaviors that foster growth and autonomy (Crant et al., 2011; Liang and Gong, 2013). Noticing the servant leader’s dedication to their development and the encouragement of their ideas, proactive employees are likely to use this nurturing environment to engage in work reflection, which can enhance their voice behavior (Kish-Gephart et al., 2009). In contrast, employees with a low level of proactive personality tend be passive recipients of the environment (Chong et al., 2021) and less likely to identify and act on opportunities. Such employees have a preference for maintaining the current state of affairs (Crant and Bateman, 2000), adhering to established work procedures and relying on their supervisor to address problems as they emerge (Crant et al., 2017). Consequently, they may not fully benefit from the inclusive and developmental behaviors of a servant leader and less likely to engage in spontaneous work reflection. Their passive attitude and reliance on external direction can hinder the effectiveness of servant leadership in fostering work reflection and subsequent voice behavior. Hence, this study hypothesizes as follows:

*Hypothesis 3*: Servant leadership and employee proactive personality will interact to influence employee work reflection, such that the relationship will be positive when there is an elevated level of employee proactive personality.

*Hypothesis 4*: Employee proactive personality plays a moderating role in the indirect relationship between servant leadership and employee voice behavior through employee work reflection, such that the relationship is positive when the level of employee proactive personality is high.

## Study 1: method

### Sample and procedures

To observe the proposed impact of servant leadership and to minimize common method variance (Podsakoff et al., 2012), a three-wave time-lagged design was used to collect data via the Internet. Specifically, a Credamo web-based survey platform was employed. Credamo and other online platforms have been shown to be reliable sources of data, attracting subjects with real work experience, and producing outcomes that are equivalent to those attained by utilizing samples from traditional sources. First, the survey’s purpose was explained to the platform managers, who were able to help contact participants and confirm their participation. Then, participants were invited to voluntarily join the online survey. High sample diversity has been shown to enhance the external validity or generalizability of the relevant study’s results (Demerouti and Rispens, 2014). In this study, 572 employees from different industries, including service, manufacturing, the financial sector, information technology, etc., expressed their willingness to participate.

Second, in order to infer the causal relationships in the hypothesized model, a unique code was generated to ensure the respondents’ anonymity, and three-phase data collection was conducted at one-month intervals, from April to July, 2023. In Phase 1 (T1), the respondents were asked to assess their perceptions of servant leadership and to self-evaluate their proactive personality; they also provided their demographic information. Demographic information collected included age, gender, education level, tenure, team scale and industry type, ensuring a comprehensive profile of the sample was captured. As stated above, 572 responses were received. In Phase 2 (T2, 1 month later), the 572 participants were asked to complete the section relating to employee work reflection; 532 responses were received, yielding a response rate of 93.0%. In Phase 3 (T3, 1 month later), the 532 employees who had participated in Phase 2 were asked to assess their voice behavior experience. At that time, 501 responses were received, yielding a response rate of 87.6%. All respondents who filled out the survey and fully answered the questionnaire received a payment of approximately 5 RMB. After deleting unmatched and incomplete questionnaires, the final and valid sample consisted of 489 participants from different industries, giving a valid response rate of 85.5%.

Of the final 489 employees, 158 (32.3%) were male, and 331 (67.7%) were female, and 364 (74.5%) had received a college or undergraduate education. Most of the respondents were from 31 to 35 (219: 44.8%) or 25–30 years old (120: 24.5%). The organizational tenure for most respondents ranged from 6 to 10 years (218: 44.6%). The manufacturing industry accounted for the largest proportion of the employees (174: 35.6%). The largest proportion of the participants worked in teams that ranged in size from 11 to 20 (210: 42.9%). The demographic profile information is depicted in Table 1.

**Table 1:** The demographic profile of the valid sample data (Study 1).

Demographic characteristics	Categories	Numbers	Percentages
Gender	Male	158	32.3
	Female	331	67.7
Age	24 and under	39	8.0
	25–30	120	24.5
	31–35	219	44.8
	36–40	66	13.5
	41–45	27	5.5
	46 and above	18	3.7
Education level	High school and below	8	1.6
	University (including junior college and bachelor)	364	74.5
	Master and above	117	23.9
Tenure	<3 years	54	11.0
	3–5 years	75	15.3
	6–10 years	218	44.6
	11–20 years	121	24.7
	>20 years	21	4.3
Team scale	4 and under	2	0.4
	5–10	88	18.0
	11–20	210	42.9
	21–49	126	25.8
	50 and above	63	12.9
Industry type	Manufacture	174	35.6
	Service	63	12.9
	Internet	86	17.6
	Financial	32	6.5
	Information technology	58	11.9
	Others	76	15.5

*N* = 489.

### Measures

The commonly used back-translation procedure was used to generate the Chinese measures for the questionnaires in this study (Brislin, 1986). A 5-point Likert scale was adopted, with values ranging from 1 = *strongly disagree/worst*, to 5 = *strongly agree/best*.

#### Servant leadership (T1)

Each employee was asked to rate his or her perceptions of servant leadership, using a seven-item scale developed by Liden et al. (2015). Example items include, “My leader makes my career development a priority,” and “My leader puts my best interests ahead of his/her own.” The Cronbach’s alpha for this scale was 0.717.

#### Employee work reflection (T2)

Similar to Leblanc et al. (2022), we measured employee work reflection with eight items from Ong et al. (2023). Example items include, “I reflect on whether I am meeting the project goals,” and “I reflect on the kind of energy I am bringing to the project.” The Cronbach’s alpha for this scale was 0.712.

#### Employee voice behavior (T3)

This was measured using the 10-item scale developed by Liang et al. (2012). Sample items include, “I proactively develop and make suggestions for issues that may influence the unit,” and “I speak up honestly with regard to problems that might cause serious loss to the work unit, even when/though dissenting opinions exist.” The Cronbach’s alpha for this scale was 0.725.

#### Employee proactive personality (T1)

This was assessed using a 10-item scale developed by Seibert et al. (1999). A representative item is, “Wherever I have been, I have been a powerful force for constructive change.” The Cronbach’s alpha for this scale was 0.733.

#### Control variables (T1)

In this paper, gender, age, education level, tenure, team scale, and industry type are used as control variables, following established precedents in the literature. Gender was a dummy variable, coded as 1 for men and 0 for women, as gender differences may influence behavior and perceptions within organizational settings (Eagly and Carli, 2009). The age variable includes six grades, reflecting the potential impact of age on employee attitude and motivation (Truxillo et al., 2015). The education level covers three grades, which correlates with job performance (Ng and Feldman, 2009). Tenure covers five grades, used to control for varying levels of experience which can affect employees’ accumulation of institutional knowledge (Ng and Feldman, 2010). Team scale covers five grades, included as larger teams may experience different dynamics and efficiency levels (Hoegl and Gemuenden, 2001). Finally, industry type covers six grades, as industry-specific factors can significantly impact employee behavior and organizational outcomes (Tabassi et al., 2019).

## Study 1: results and discussion

### Descriptive statistics and analysis

Table 2 presents the descriptive statistics, intercorrelations, and scale reliabilities for the study’s variables. As shown in Table 1, the total Cronbach’s α coefficients of all scales are greater than 0.70, indicating that each scale has good internal consistency. The correlation matrix shows that servant leadership was found to be positively related to employee work reflection (*r* = 0.634, *p* < 0.01), and that employee work reflection was found to be positively related to employee voice behavior (*r* = 0.670, *p* < 0.01). These findings are is in line with prior theorizing on SCT Furthermore, consistent with our SCT framework, servant leadership was found to be positively related to employee voice behavior (*r* = 0.710, *p* < 0.01).

**Table 2:** Means, standard deviations, and correlations among the variables (Study1).

Variables	*M*	*SD*	1	2	3	4	5	6	7	8	9	10
1. Sex (T1)	0.320	0.468	–									
2. Age (T1)	2.950	1.119	0.030	–								
3. Education (T1)	3.170	0.560	−0.108^*^	−0.055	–							
4. Tenure (T1)	2.960	1.007	−0.024	0.811^**^	−0.039	–						
5. Team scale (T1)	3.330	0.930	0.049	0.122^**^	−0.005	0.187^**^	–					
6. Industry type (T1)	3.800	3.045	−0.040	−0.008	−0.164^**^	−0.013	−0.118^**^	–				
7. Servant leadership (T1)	4.267	0.449	−0.042	0.243^**^	0.070	0.283^**^	0.045	−0.058	**0.717**			
8. Employee work reflection (T2)	4.402	0.359	−0.048	0.164^**^	0.078	0.206^**^	0.089^*^	−0.092^*^	0.634^**^	**0.712**		
9. Employee voice behavior (T3)	4.337	0.351	0.046	0.259^**^	0.068	0.328^**^	0.080	−0.020	0.710^**^	0.670^**^	**0.725**	
10. Employee proactive personality (T1)	4.354	0.350	−0.011	0.228^**^	0.092^*^	0.285^**^	0.081	−0.033	0.627^**^	0.652^**^	0.725^**^	**0.733**

*N* = 489. **p < 0.01, *p < 0.05. The bold values in this table represent the reliability coefficients for the corresponding scales.

### Confirmatory factor analysis and descriptive statistics

To assess the appropriateness of the measurement model, including the five studied variables, a confirmatory factor analysis (CFA) using Mplus 8.3 software was first performed to assess the model fit (Muthén and Muthén, 2012). The fit indices involved a comparative fit index (CFI), Tucker–Lewis index (TLI), and root mean square residual (RMSEA). All these methods have been commonly used to evaluate various aspects of model fit and have been reported in related organizational literature (Brown, 2006). As indicated in Table 3, the results show that the hypothesized four-factor model had an adequate fit [*χ*^2^ (504) = 836.798, TLI = 0.920 > 0.90, CFI = 0.932 > 0.90, RMSEA = 0.037 < 0.05, SRMR = 0.046 < 0.08] and was better than other alternative models. Therefore, the focal constructs are distinct in this study.

**Table 3:** The results of confirmatory factor analyses (Study1).

Models	Variable combination approaches	*χ^2^*	*df*	*χ^2^/df*	CFI	TLI	RMSEA	SRMR
Four-factors model	SL, EWR, EVB, EPP	836.798	504	1.660^***^	0.932	0.920	0.037	0.046
Three-factors model	SL, EWR + EVB, EPP	947.911	507	1.870^***^	0.910	0.894	0.042	0.047
Two-factors model	SL, EWR + EVB + EPP	996.286	509	1.957^***^	0.900	0.884	0.044	0.048
Single-factor model	SL + EWR + EVB + EPP	1053.392	510	2.065^***^	0.889	0.871	0.047	0.048

*N* = 489. SL, servant leadership; EWR, employee work reflection; EVB, employee voice behavior; EPP, employee proactive personality; CFI, comparative fit index; TLI, Tucker–Lewis index; SRMR, standardized root mean square residual.^***^p < 0.001.

### Hypothesis testing

**Figure 2:**
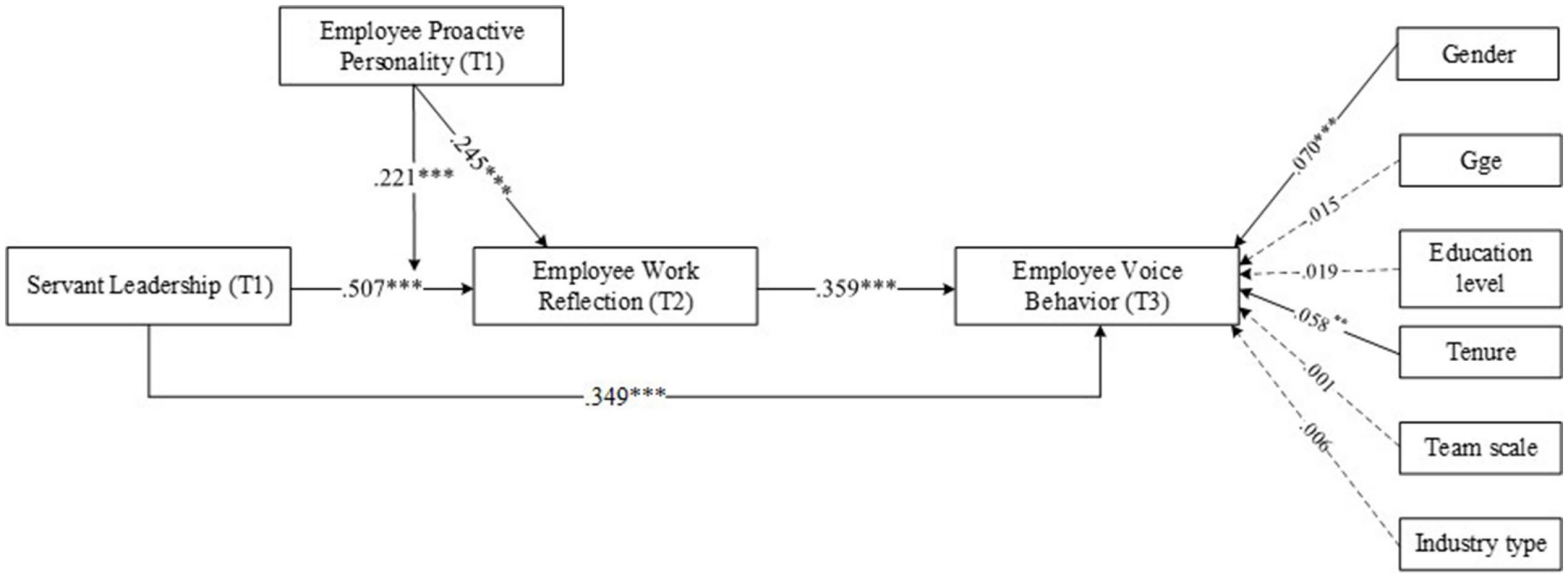
Results of the modeling analysis.

Modeling path analysis and Monte Carlo simulation were conducted through Mplus and R package to test the mediation and moderation hypotheses, with confidence intervals. A total of 20,000 estimation times are recommended when using the Monte Carlo bootstrapping method (Hayes, 2015), and Table 4 presents the result of hypotheses testing and Figure 2 summarizes the hypothesis model. As shown in Figure 2, the direct effects of servant leadership on employee voice behavior is significant (*β* = 0.349, *p* < 0.001). Thus, Hypothesis 1 is supported. Specifically, servant leadership exhibited a significant positive relationship with employee work reflection (*β* = 0.507, *p* < 0.001). Furthermore, employee work reflection was positively related to employee voice behavior (*β* = 0.359, *p* < 0.001). Referring to Table 4 and Figure 2, the mediating effect of employee work reflection between servant leadership and employee voice behavior was found to be significant (*β* = 0.228, *p* < 0.001, *SE* = 0.027, 95% CI [0.177; 0.281], Model 2). Thus, Hypothesis 2 is supported.

**Table 4:** Results of path analysis and Monte Carlo bootstrapping confidence intervals (Study1).

Models and effects	Effect	*SE*	MC CIs	
			95% LL	95% UL
Direct Effect (Model 1)				
Servant leadership → Employee voice behavior	349^***^	0.052	0.255	0.460
Servant leadership → Employee work reflection	0.507^***^	0.088	0.333	0.674
Employee work reflection → Employee voice behavior	0.359^***^	0.098	0.166	0.545
Mediation Effect (Model 2)				
Servant leadership → Employee work reflection → Employee voice behavior	0.228^***^	0.027	0.177	0.281
Moderating Effect (Model 3) on “Servant leadership → Employee work reflection” Relationship				
High employee proactive personality (M + 1SD)	0.768^***^	0.040	0.690	0.846
Low employee proactive personality (M-1SD)	0.473^***^	0.039	0.396	0.551
Difference	0.295^**^	0.029	0.239	0.351
Moderated Mediating Effect (Model 4) on “Servant leadership → Employee work reflection → Employee voice behavior” Relationship				
High employee proactive personality (M + 1SD)	0.282^***^	0.032	0.221	0.347
Low employee proactive personality (M−1SD)	0.174^***^	0.023	0.131	0.220
Difference	0.108^***^	0.015	0.080	0.140

*N* = 489.SE, standard error. Bootstrap = 20,000. **p < 0.01; ***p < 0.001.

**Figure 3:**
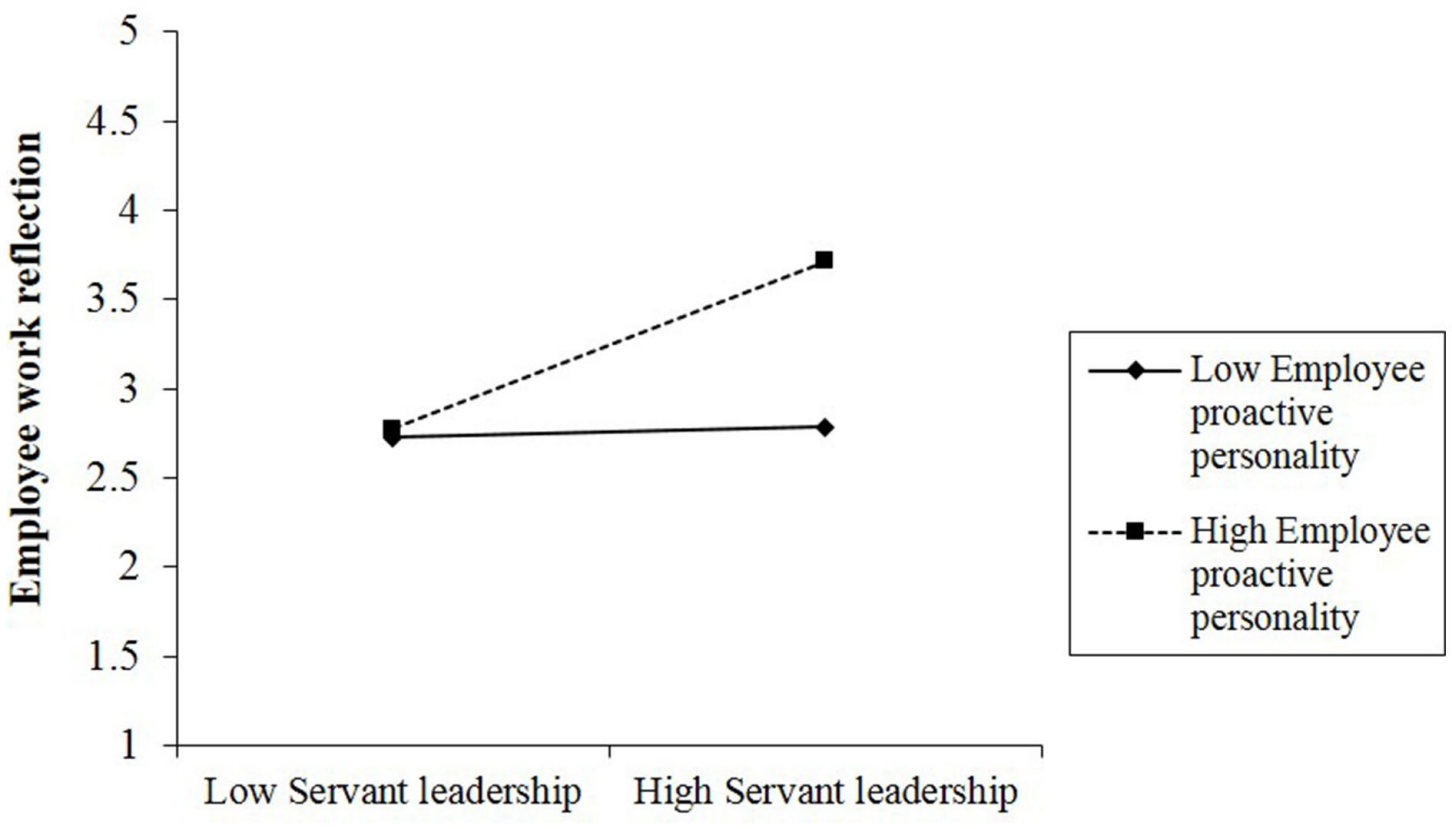
The interactive effect of servant leadership and employee proactive personality on employee work reflection (Study 1).

Hypothesis 3 proposes that employee proactive personality accentuates the relationship between servant leadership and employee work reflection. As indicated in Figure 2, the moderating effect of proactive personality was significant (*β* = 0.221, *p* < 0.001). According to the method of Aiken and West (1991), the significant moderating effects were plotted in Figure 3 using the moderator at high (one standard deviation above the mean value) and low (one standard deviation below the mean value) levels. Furthermore, the significance of the simple slope at high and low levels was examined using the bootstrapping method with Monte Carlo simulation. When employee proactive personality was high, servant leadership was found to have a significant effect on employee work reflection (effect = 0.768, *p* < 0.001, *SE* = 0.040, 95% CI [0.690; 0.846], Model 3). Under the condition of low employee proactive personality, the relationship was also found to be significant (effect = 0.473, *p* < 0.001, *SE* =0.039, 95% CI [0.396; 0.551], Model 3). The difference between these two slopes was also significant (Δeffect = 0.295, *p* < 0.001, SE = 0.029, 95% CI [0.239; 0.351], Model 3). Thus, Hypothesis 3 is supported.

The results presented in Table 4 also support the moderated mediation effects. The mediating effect of leader identity between servant leadership and employee voice behavior under the condition of employee proactive personality was found to be significant (Δeffect = 0.108, *p* < 0.001, *SE* = 0.015, 95% CI [0.080; 0.140], Model 4). Thus, Hypothesis 4 is supported.

Despite the multi-time point design, Study 2 further explored the moderating effect of employee proactive personality in an experiment to establish a clearer causal relationship between servant leadership and employee work reflection. This approach allowed for a focused examination of how servant leadership and proactive personality interact to influence work reflection (Hypothesis 3).

## Study 2: method

### Participants

To complete Study 2, we recruited 107 full-time employees from China, all with prior team experience under supervision, via the Credamo platform. The appropriate sample size for Study 2 was estimated using the G*power (Erdfelder et al., 1996). Given the two-factor between-subjects experimental design, the following parameters were specified: an *F*-test with ANOVA for fixed effects, special, main effects, and interactions, an effect size *f* of 0.40 (reflecting a medium to large effect size based on Cohen’s conventions), an alpha level (*α*) of 0.05, and a desired power (1-*β*) of 0.80. The numerator degrees of freedom were set to 1, and the number of groups was set to 4, corresponding to the 2×2 factorial design (Clemente et al., 2020). The results of G*power show a minimum sample size of 52 participants is required to achieve statistically significant results, which is much smaller than the actual sample size of this study (*N* = 107).

These employees represented a diverse range of industries including IT, biopharmaceuticals, product manufacturing, engineering, chemical engineering, and procurement. Participants who joined the experiment through Credamo were compensated with 2 RMB. Further details about the sample include 52 (48.6%) were male, and 55 (51.4%) were female, and 90 (84.1%) had received a college or undergraduate education. Most of the respondents were from 26 to 30 (30: 28.0%) or 31–40 years old (35: 32.7%). The organizational tenure for most respondents ranged from 6 to 10 years (27: 25.2%). The information technology industry accounted for the largest proportion of the employees (32: 29.9%).

### Procedure and experimental design

Both servant leadership and employee proactive personality were manipulated, leading to 2 (servant leadership: high vs. low) × 2 (employee proactive personality: high vs. low) between-subjects design. The participants were randomly allocated to one of the four experimental conditions. Twenty-eight individuals were assigned to the condition of high servant leadership, with high employee proactive personality; 26 participants were assigned to the condition of high servant leadership and high employee proactive personality; 26 participants, and 27 participants, respectively, were assigned to each of the other two conditions. Employee work reflection was the dependent variables. Participants were informed that their task in the experiment involved reading a concise, hypothetical scenario and answering a series of questions related to it.

Across all four experimental conditions, the fundamental scenario description remained consistent. Participants were instructed to assume the role of an employee at a reputable general consulting firm, where the predominant work style involves functioning primarily as a project team. The team comprises the participant’s supervisor, Li Yang, and three other team members (including Participants). Since their appointment, they have encountered the following situations in their work environment.

### Manipulation

For parsimony, manipulation of servant leadership and employee proactive personality are demonstrated in Appendix A.

### Measures

#### Servant leadership

To assess perceptions of Li Yang’s servant leadership, the participants were asked 7 items developed by Liden et al. (2015). A sample item is “Li Yang puts my best interests ahead of his/her own.” The Cronbach’s *α* = 0.891.

#### Employee proactive personality

To assess participants’ level of employee proactive personality, they were asked seven items developed by Seibert et al. (1999). Sample items include, “Wherever I have been, I have been a powerful force for constructive change.” The Cronbach’s *α* = 0.957.

#### Employee work reflection

The scale (Ong et al., 2023) adopted in Study 1 was also used to assess employee work reflection. A sample item of fixed mindset is, “I reflect on whether I am meeting the project goals.” The Cronbach’s alpha of coworker malicious envy was 0.951.

#### Control variables

The same control variables were used as those of Study 1: gender, age, education level, industry type.

### Study 2: results and analysis

#### Manipulation checks

To test whether the manipulation of servant leadership vs. employee proactive personality was successful, participants were asked to “Based on the above scenario, please judge to what extent the supervisor on the team, Li Yang, exhibits servant-leader behaviors?” “Based on the above scenario, please determine the extent to which I on the team exhibit a proactive personality?” Both items were assessed on a 5-point scale (from 1 = not at all to 5 = very much). An analysis of the variance results show that the servant leadership manipulation had a strong effect on the servant leadership check (*F* = 817.836, *p* < 0.001, *η*^2^ = 1.665). Similarly, the employee proactive personality manipulation had a strong effect on the employee proactive personality check (*F* = 535.724, *p* < 0.001, *η*^2^ = 2.348). These results indicate that our manipulations were successful.

### Intercorrelations of the measures

The calculation of the intercorrelations showed that servant leadership correlated with employee proactive personality; *r* = 0.225, *p* < 0.05, and employee proactive personality correlated with employee work reflection, *r* = 0.799, *p* < 0.01. The observed correlations suggest that the investigated constructs are correlated, yet they do not demonstrate a high degree of similarity. Consequently, they exhibit adequate discriminant validity. Table 5 displays the correlation coefficients of the variables.

**Table 5:** Means, standard deviations, and correlations among the variables (Study 2).

Variables	Mean	*SD*	1	2	3	4	5	6	7	8
1.Sex	0.486	0.502	–							
2.Age	2.430	1.056	−0.095	–						
3.Education	2.907	0.637	−0.004	−0.080	–					
4.Tenure	2.243	1.106	−0.215^*^	0.733^**^	−0.061	–				
5.Industry type	3.551	2.020	0.031	−0.125	0.033	−0.124	–			
6.Servant leadership	3.449	1.039	0.097	0.060	−0.099	0.083	0.009	**(0.891)**		
7.Employee proactive personality	3.456	1.040	0.119	0.034	0.034	0.036	0.153	0.225^*^	**(0.957)**	
8.Employee work reflection	3.494	1.010	0.166	0.018	0.069	0.049	0.173	0.171	0.799^**^	**(0.951)**

*N* = 107.***p < 0.001, **p < 0.01, *p < 0.05.

### Hypotheses testing

**Figure 4:**
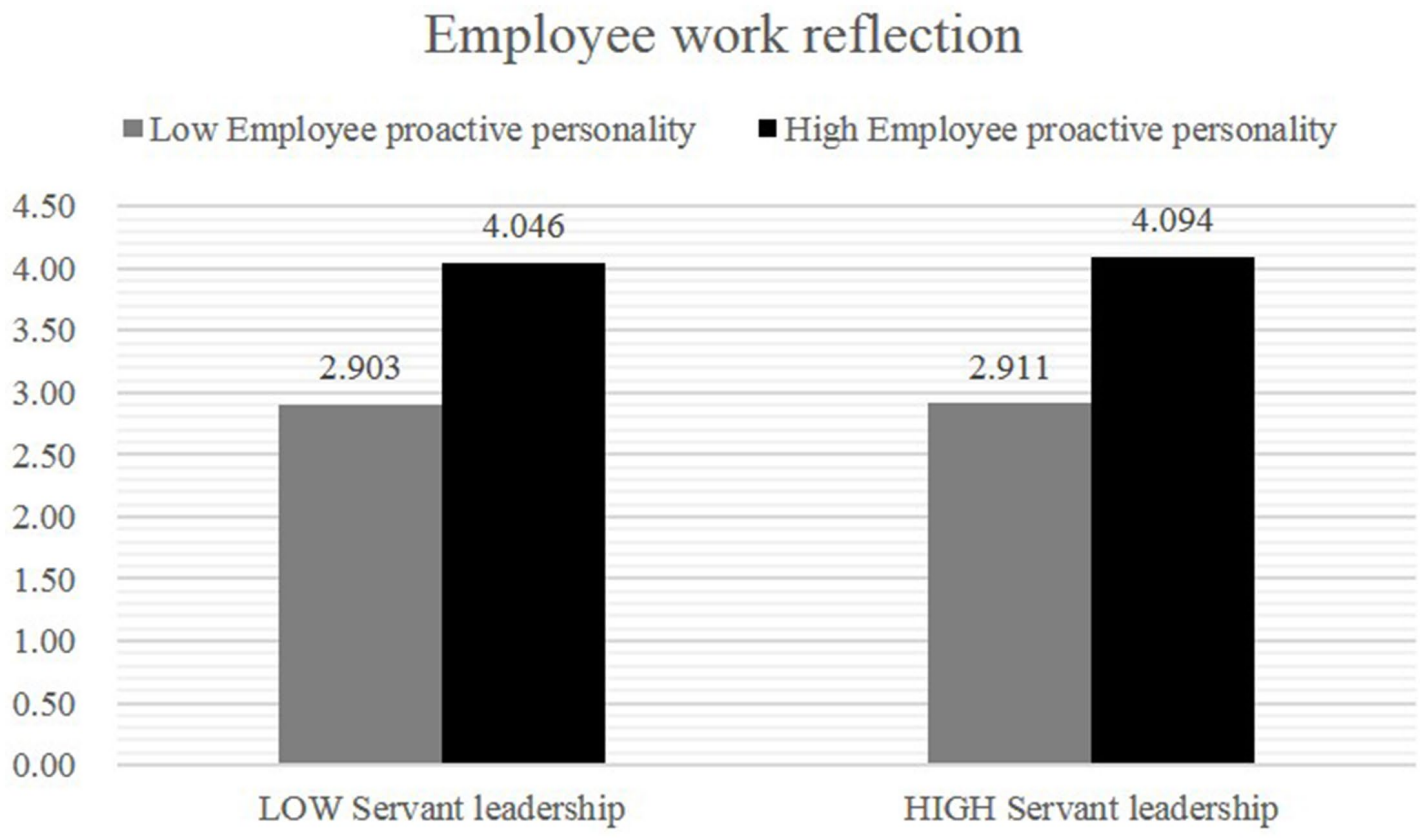
The interactive effect of servant leadership and employee proactive personality on employee work reflection (Study 2).

Hypothesis 3 predicts an interactive effect of servant leadership and employee proactive personality on employee work reflection. The analysis of the variance results suggest that the interactive effect of servant leadership and employee proactive personality on employee work reflection was significant, *F = 3.249, p < 0.05, η^2^ = 0.314*. The direction of the interaction effect was in the hypothesis direction (Figure 4). Planned comparisons show that, in the low employee proactive personality condition, the levels of employee work reflection were significantly higher in the high servant leadership condition (*M = 2.911, SD = 1.03*) than in the low servant leadership condition (*M = 2.903, SD = 1. 056*). Moreover, when participants were in the high employee proactive personality condition, those in the high servant leadership felt a significantly higher level of employee work reflection (*M = 4.094, SD = 0.626*) than in the low servant leadership condition (*M = 4.046, SD = 0.340*).

In sum, through an experimental manipulation, Study 2 provides strong evidence of the interactive effects of servant leadership and employee proactive personality on employee work reflection. Results from the independents samples demonstrate that the relationship between servant leadership and employee work reflection is stronger when employee proactive personality is high than when it is low. Hence, Hypothesis 3 was supported.

## General discussion

### Theoretical implications

This study contributes to servant leadership literature in several ways. Firstly, a novel theoretical framework is presented that integrates the servant leadership and employee voice literatures by introducing employee work reflection as individual cognitive process. While previous research has shown that servant leadership promotes voice behavior through modeling and interaction (Detert and Treviño, 2010; Arain et al., 2019; Liao et al., 2021; Hartnell et al., 2023), the cognitive mechanism linking servant leadership and employee voice behavior has remained unclear. This study highlights how internal employee reflection processes mediate the influence of servant leadership on employee voice behavior, focusing on understanding internal controllable factors and the external environment.

Secondly, this research supports the view that the efficacy of servant leadership depends on individual factors (Hartnell et al., 2023). It highlights the significant moderating role of employee proactive personality, addressing calls for elucidation of individual traits within the servant leadership process (Arain et al., 2019). The findings indicate that servant leadership may not inspire voice behaviors in employees lacking proactive personality. This underscores the importance of considering individual differences when evaluating servant leadership, thus expanding the existing literature by incorporating individual inclinations within the SCT framework.

Finally, this study integrates SCT with existing servant leadership literature. Previously, servant leadership and employee positive extra-role behavior research has leaned largely on social exchange theory (e.g., Mayer, 2010). Based on SCT, this study argues that servant leadership stimulates employee voice behavior by encouraging employees to engage in work reflection. This broadens the scope of servant leadership’s impact by illustrating its indirect effect on employee voice behavior, highlighting the importance of cognitive and personality factors in this dynamic.

### Practical implications

The findings of this research provide some practical implications. First, the results highlight the crucial role of employee work reflection in promoting employee voice behaviors. Organizations should encourage work reflection by guiding employees through action reviews or organizing reflection sessions. Inducing work reflection through carefully selected methods can enhance employee voice behavior.

Another key finding is that servant leadership is more salient for employees with a high level of proactive personality. Organizations should consider proactive personality as a significant moderator of employees’ response to servant leadership. For proactive employees, servant leadership enhances motivation to voice. To improve decision-making and organizational learning, organizations should identify proactive individuals and allocate resources to foster their ability to voice.

Finally, this study highlights the value of servant leadership in organizations. Servant leaders encourage high levels of employee voice behavior. Therefore, organizations should implement training initiatives to enhance leaders’ understanding and practice of servant leadership. Additionally, fostering an environment that promotes and incentivizes servant leadership behaviors is recommended.

### Limitations and future research directions

Despite the significant theoretical and practical implications, this study also has some limitations. First, employee proactive personality was only examined as a moderator, and employee work reflection was only examined as a mediator. Future research should examine other individual moderators (such as political skill) and other potential mediators (such as psychological safety). This is because employees with high levels of political skill are better able to navigate organizational dynamics and build relationships, which can enhance their voice behaviors. Similarly, a psychologically safe environment encourages employees to express their ideas and concerns without fear of negative consequences. Exploring these factors would provide a more comprehensive understanding of the influences on employee behavior.

Second, the specific cultural and organizational context of the sample further limits the generalizability of the findings. Future research should replicate this study in different cultural and organizational settings to determine whether the findings hold across various contexts. This would enhance the external validity of the study and provide a more comprehensive understanding of the dynamics at play in diverse environments.

Third, this study may not fully eliminate common method bias, as all assessments were self-reported by employees. To mitigate this bias, this study employed time-lagged questionnaires and utilized scales that had been previously validated. However, to further address the potential impact of self-reporting bias and enhance the robustness of the conclusions, we suggest that future studies should gather data from additional sources such as peer assessments, supervisory ratings, or objective performance indicators. These measures could help to reduce the reliance on self-reported data and provide a more comprehensive view of the variables involved.

Fourth, in Study 1, more than 50% of the participants were female, gender has been shown to correlate with employee voice behavior. Exploring how gender and other demographic variables influence the effectiveness of servant leadership could provide deeper insights into the dynamics at play. We recommend that future research explore the data associated with participant characteristics such as gender.

## Conclusion

This research contributes to servant leadership literature by empirically investigating how and when servant leadership promotes employee voice. Based on a three-wave time survey and one experimental study, the findings of this study indicate that the mediating role of employee work reflection is significant in this particular relationship. In addition, employee proactive personality is found to act as a moderating factor. We anticipate further investigation into the mechanisms by which servant leadership is associated with employee outcomes, as well as the discovery of other factors and contextual limitations that influence the effectiveness of servant leadership.

## Data availability statement

The raw data supporting the conclusions of this article will be made available by the authors, without undue reservation.

## Ethics statement

The studies involving humans were approved by Ethics Committee of Jilin University. The studies were conducted in accordance with the local legislation and institutional requirements. The participants provided their written informed consent to participate in this study.

## Author contributions

ZX: Conceptualization, Data curation, Formal analysis, Investigation, Methodology, Software, Supervision, Validation, Writing – original draft, Writing – review & editing. YG: Conceptualization, Data curation, Formal analysis, Investigation, Methodology, Software, Validation, Writing – original draft. HW: Conceptualization, Funding acquisition, Resources, Supervision, Validation, Visualization, Writing – review & editing. LL: Conceptualization, Investigation, Project administration, Resources, Validation, Visualization, Writing – review & editing.
