# Supplementary material for: Servant leadership and employee voice behavior: the role of employee work reflection and employee proactive personality
Source: Front Psychol. 2024 Jul 29;15:1421412. doi: 10.3389/fpsyg.2024.1421412 (PMC11317245; doi:10.3389/fpsyg.2024.1421412)
Supplement: Supplementary file 1 [file Table_1.DOCX]

**Appendix A. Experimental Materials of Study 2**

**Manipulation**

For parsimony, manipulation of servant leadership and employee proactive personality are demonstrated in Appendix A.

Then, the manipulation of leader-member exchange was introduced, based on the previous research of Wu et al. (2021) and the servant leadership scale by Liden et al. (Liden et al., 2015). In the ***high servant leadership*** condition, the scenario said:

*Xiao Wang’s father broke his arm and requires additional assistance throughout the recovery weeks. Your supervisor, Li Yang, has granted Xiao Wang the flexibility to work during this period. Four months ago, a promising new project emerged* *that you knew would be offer valuable career experience. However, you were engaged in another project aligned with your current skills. Despite the potential slowdown in pace while acquiring new skills, you requested Li Yang to transfer you to the new project. It is gratifying, though not unexpected, that your request is approved, as it aligns with the common approach of your team in managing such inquiries. Shortly after you begin work on the new project, your team encounters an unexpected challenge that has the potential to delay the completion date. Although the project is highly visible, your team has not yet fully understood the fact that the project is experiencing a major problem. Li Yang quickly realizes that the project is in trouble, but despite this, Li Yang still has confidence in the team, empowers the team and finds a solution to the problem. Again, you are not surprised because this is exactly what your team trusts and expects from Li Yang. Over the past few months, Li Yang faced two opportunities to attain short-term goals through ethically questionable decisions. In both cases, he unequivocally declined to breach any ethical principles, serving as a positive example for the team. Beyond his regular duties, Li Yang motivates each team member to dedicate time to volunteerism, contributing to the community, even if these activities aren’t officially tied to company programs.*

In the ***low servant leadership*** condition, the scenario said:

*Your supervisor, Li Yang, is usually fair, but makes decisions that are designed to maximize upper management’s view of the team and Li Yang’s productivity. Four months ago, a new project emerged that you knew would be offer a promising career experience. However, you were engaged in another project more aligned with your current skills. Although you requested Li Yang to transfer you to the new project, Li Yang advised you to remain on the current project. Li Yang expressed concern that learning the skills for the new project might impede your work pace, resulting in a loss for the company. Recently, your team has faced some work-related challenges that could significantly impede the project schedule. Initially, Li Yang was unaware of the issues, but after you elucidated the problems and proposed solutions, Li Yang declined to allow you to address the problems autonomously, instructing you on the course of action. As part of the resolution, Li Yang provides inaccurate information to the client regarding what your team provided. Although this strategy ensured meeting the project deadline, you find it inconsistent with the ethics training regularly provided. Besides your routine tasks, you aspire to engage in volunteer activities contributing to the community. Regrettably, Li Yang opposes this, asserting that such volunteer services lack relevance to the company's official programs.*

Following that, the employee proactive personality scenarios were introduced. In the ***high employee proactive personality*** condition, participants read:

*When a problem arises in the team, I promptly engage in collective discussions to address it positively, seeking solutions. I capitalize on opportunities to distribute workload between two colleagues, aiming to alleviate the burden. Simultaneously, to ensure timely completion of production tasks, I consistently take the initiative to shoulder tasks beyond my assigned responsibilities. I strive to propose innovative project solutions to enhance work efficiency and promptly implement new ideas.*

In the ***low ethical leadership*** *condition*, participants read:

*When problems occurred in the team, I exhibited passivity and failed to initiate discussions. I overlooked the assignment as an opportunity to enhance the training process and neglected to proactively propose ways to distribute the workload without disrupting the work schedule. Even when confronted with the potential non-completion of a task, I limited myself to completing the essential daily tasks, showing reluctance to take on additional responsibilities. I also refrained from devising novel project solutions to enhance work efficiency.*
